# Clinical Features and Neurodevelopmental Outcomes for Infants with Perinatal Vertical Transmission of Zika Virus, Colombia

**DOI:** 10.3201/eid2802.204551

**Published:** 2022-02

**Authors:** Luis A. Pérez-Vera, Valentina Herrera-García, María C. Pérez-Matos, Luis A. Díaz-Martínez, Luis A. Villar-Centeno, Luz S. Pinilla-García, Mario A. Rojas

**Affiliations:** Hospital Universitario de Santander, Bucaramanga, Colombia (L.A. Pérez-Vera);; Universidad Industrial de Santander, Bucaramanga (L.A. Pérez-Vera, V. Herrera-García, L.A. Díaz-Martínez, L.A. Villar-Centeno, L.S. Pinilla-García, M.A. Rojas);; Harvard School of Public Health, Boston, Massachusetts, USA (M.C. Pérez-Matos)

**Keywords:** Zika virus, viruses, neurodevelopment, clinical features, perinatal infection, infants, newborns, vertical transmission, vector-borne infections, Colombia

## Abstract

Transplacental transmission of Zika virus has been reported during all trimesters of pregnancy and might lead to central nervous system anomalies, including microcephaly. We report 3 cases of perinatal Zika infection identified during the epidemic in Colombia and provide detailed descriptions of clinical features, diagnosis, and neurodevelopmental outcome at 18 months of age (corrected).

The emergence of Zika virus (ZIKV) in the Americas has coincided with an abnormal increase in prenatal and neonatal documented cases of microcephaly and other anomalies of the central nervous system (*1*) These alterations of the brain, along with animal models of vertical transmission of ZIKV, a single-stranded RNA flavivirus, are evidence of the neurotropic nature of the virus (*2**–**4*).

Vertical transmission and infection of the fetus during all 3 trimesters of pregnancy with ZIKV has been extensively reported, but little is known about perinatal transmission; only a few cases have been reported (*5*–*8*). We report 3 cases of perinatal ZIKV infection during the epidemic of Zika in Colombia and data on the neurodevelopmental outcome at 18 months of age (corrected).

## The Study

The Institutional Review Board and Ethics Committee of the Universidad Industrial de Santander approved this study. Formal written consent was obtained from participating women.

Case-patient 1 was a 26-year-old pregnant woman in labor who was admitted to the Hospital Universitario de Santander on August 7, 2015, after she reported fever, exanthema (maculopapular rash on the torso), and osteoid‒muscular pains (Figure). Her initial hemogram showed mild thrombocytopenia (112,000 platelets/μL; reference range 150,000‒400,000 platelets/μL). Test results for syphilis, toxoplasmosis, rubella, cytomegalovirus, herpes simplex virus, varicella zoster virus, and parvovirus B19 during hospitalization were negative, as was a test result for dengue virus (DENV) IgM.

**Figure:**
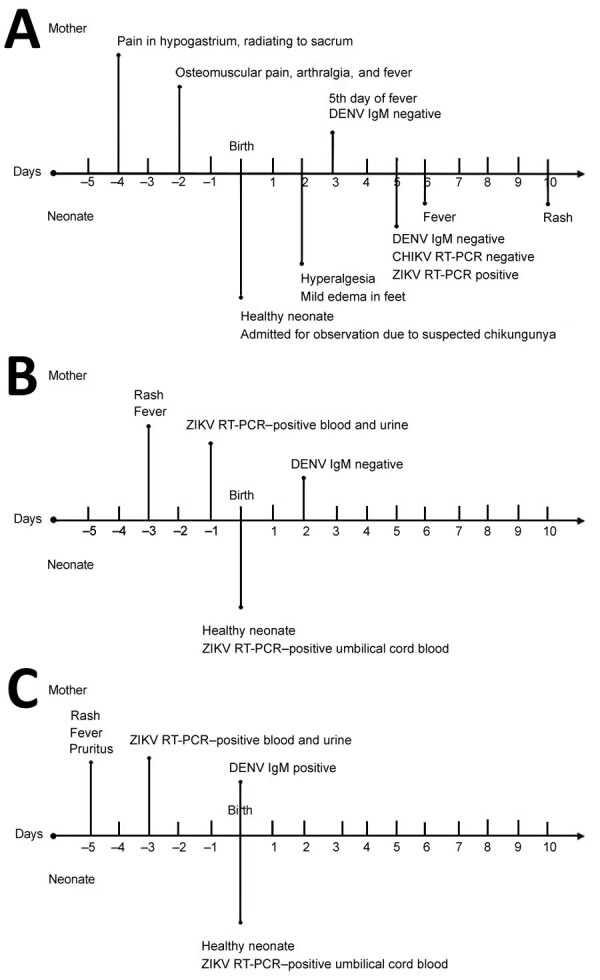
Timelines for 3 case-patients who had perinatal Zika virus infection during a Zika epidemic, Colombia, 2015. A) Case-patient 1; B) case-patient 2; C) case-patient 3. DENV, dengue virus; CHIKV, chikungunya virus; RT-PCR, reverse transcription PCR; ZIKV, Zika virus.

A boy was born vaginally at 37 weeks of gestation. Apgar scores were normal. Anthropometric measurements yielded a birthweight of 3.03 kg (32nd percentile), a head circumference (HC) of 32 cm (6th percentile), and a length of 54 cm (99th percentile). Results of a physical examination were unremarkable. The infant was admitted for observation, and cord blood samples were sent to the Instituto Nacional de Salud (INS) for additional testing by reverse transcription PCR (RT-PCR) for DENV and chikungunya virus. On the second day of life, the infant had a distal macular-papular rash with hyperalgesia and mild edema of the hands and feet; a test result for DENV IgM was negative. Hyperthermia developed on the sixth day, and generalized exanthema developed on the seventh day.

Because of persistent fever, we initiated a sepsis work-up. Results for initial complete blood count/differential count, erythrocyte sedimentation rate, blood culture, urine culture, and C-reactive protein were within reference ranges. Fever and exanthema were present for 4 additional days; a follow-up C-reactive protein level was abnormal (21 mg/dL). Findings for analyses of cerebrospinal fluid and culture were unremarkable. After symptoms improved, the infant was discharged; an RT-PCR result from the INS was negative for chikungunya virus.

Because of the Zika epidemic in Brazil, the INS initiated RT-PCR testing for ZIKV and randomly retested samples from epidemiologic surveillance of DENV and chikungunya virus (*9*). On January 20, 2016, the INS reported that the RT-PCR result for ZIKV for the infant was positive. Neuroimaging was not indicated. On April 3, 2017, a follow-up was conducted when the child was 19.3 months of age (corrected). Anthropometric measurements showed a weight of 10.6 kg (21st percentile), a height of 85 cm (85th percentile), and an HC of 46 cm (35th percentile). Results of audiology and ophthalmologic evaluations were unremarkable. At 21.4 months of age (corrected), we conducted a neurodevelopmental evaluation by using the Bayley Scale for Infant and Toddler Development (*10*); both gross and fine motor scored at the 50th percentile, cognition at the 75th percentile, and expressive and receptive language at the 18th percentile (Table).

**Table:** Neurodevelopmental evaluation with the Bayley Scale for Infant and Toddler Development at each visit for 3 case-patients who had perinatal Zika virus infection, Colombia*

Case-patient	Infant age, mo	Cephalic perimeter Z score	Scale domain										
			Motor				Language				Cognitive		
			Score†	Z score	Percentile		Score†	Z score	Percentile		Score†	Z score	Percentile
1	20.1	−0.50	94	−0.40	34		94	−0.40	34		90	−0.67	25
	24.9	−1.70	107	0.47	68		83	−1.13	13		100	0.00	50
	40.3	−0.51	88	−0.80	21		89	−0.73	23		95	−0.33	37
2	7.2	0.86	100	0.00	50		NT	NT	NT		NT	NT	NT
	13.0	0.64	97	−0.20	42		97	−0.20	42		85	−1.00	16
	36.5	0.21	110	0.67	75		91	−0.60	27		90	−0.67	25
3	8.4	0.72	115	1.00	84		NT	NT	NT		NT	NT	NT
	14.5	0.89	112	0.80	79		94	−0.40	34		110	0.67	75
	21.6	0.52	100	0.00	50		89	−0.73	23		110	0,67	75

*NT, not tested. †Standardized.

Case-patient 2 was a 30-year-old pregnant woman who was admitted to the Hospital Universitario de Santander at 38 weeks of gestation with a history of unspecified discomfort, musculoskeletal pain, and fever (temperature 38°C) 2 days before admission. She had a history of thrombocytopenia during gestation. Her admission platelet count was 80,000/μL. Results for *Toxoplasma gondii* IgG and IgM, HIV, hepatitis B surface antigen, and venereal disease research laboratory testing were negative. Test results of blood and urine samples by RT-PCR for ZIKV were positive. On the fifth day of maternal symptoms, a test result for DENV IgM was positive, suggesting co-infection with both types of arbovirus. Misoprostol was then administered.

The following day, a boy was delivered vaginally. He had good Apgar scores and was sent to the nursery for observation. He had a birthweight of 3.08 kg and an HC of 35 cm, and his physical examination was uneventful. An umbilical cord blood sample was positive by for RT-PCR for ZIKV. The neonate remained asymptomatic, and both hearing and ophthalmologic evaluations were normal. Neurodevelopmental evaluation with the Bayley Scale for Infant and Toddler Development (*10*) at 7.2, 13.0, and 36.5 months of age for CA was considered within the normal range for age (Table). Neuroimaging was not indicated.

Case-patient 3 was a 25-year-old pregnant woman who was admitted to the Hospital Universitario de Santander hospital on April 5, 2016, at 34 weeks of gestation, with a history of eye irritation, rash, and fever. Test results for syphilis, toxoplasmosis, varicella zoster virus, parvovirus B19, rubella, cytomegalovirus, and herpes simplex virus were negative, but blood and urine RT-PCR results for ZIKV positive. Betamethasone was administered for lung maturation.

On the third day of admission, a boy was delivered vaginally. He had normal Apgar scores and a birthweight of 2.24 kg, an HC of 31 cm, and a length of 42 cm. An umbilical cord blood sample tested by RT-PCR for ZIKV was positive. The infant showed signs of respiratory distress syndrome at birth, requiring supplemental oxygen, ventilatory support with continuous positive airway pressure, and surfactant administration. Symptoms improved after 24 hours and he was subsequently discharged.

We conducted neurodevelopmental follow-up at 6, 12, 24, and 36 months of age by using the Bayley Scale for Infant and Toddler Development (*10*). All evaluations were considered within the normal range for age (corrected). Ophthalmologic and hearing assessments were normal; neuroimaging was not indicated.

## Conclusions

The symptoms and diagnostic tests for the 3 pregnant women strongly support maternal infection 5 days before delivery. Vertical transmission is shown by suggestive early neonatal symptoms and a positive RT-PCR result for ZIKV for case-patient 1 and RT-PCR Zika virus‒positive cord blood for the other 2 case-patients (Figure). Case-patient 2 had a positive RT-PCR result for ZIKV in blood and urine and a positive result for DENV IgM, suggesting co-infection.

Although cross-reactivity in an RT-PCR for ZIKV and other arbovirus infections is possible (*11*), co-infection with 2 different types of arbovirus is also possible because of the endemic nature of dengue in Colombia where the patients were identified (*12*). Vertical transmission of DENV is considered a rare event, and there have been no reports of congenital dengue infection in neonates born to mothers infected early during pregnancy (*13*). Case-patient 3 had a positive result by RT-PCR for ZIKV in blood and urine, and her infant was positive for ZIKV in cord blood, which enabled us to confirm vertical transmission. However, because testing for DENV or chikungunya virus was not available, we cannot speculate on this issue.

Adverse fetal central nervous system anomalies with maternal ZIKV infection have been reported during the third trimester of pregnancy (*14*,*15*). Neurodevelopmental follow-up results were uneventful for the 3 case-patients we describe who had perinatal vertical transmission. Two previous cases of perinatal transmission reported showed no evidence of neurodevelopmental impairment, thus supporting our findings (*8*). In summary, our findings indicate that perinatal infection within the time frame described for these case-patients does not appear to affect neurodevelopmental outcomes of the newborns.

## References

[1] Mlakar J, Korva M, Tul N, Popović M, Poljšak-Prijatelj M, Mraz J, et al. Zika virus associated with microcephaly. N Engl J Med. 2016;374:951–8. 10.1056/NEJMoa1600651 26862926

[2] Tang H, Hammack C, Ogden SC, Wen Z, Qian X, Li Y, et al. Zika virus infects human cortical neural progenitors and attenuates their growth. Cell Stem Cell. 2016;18:587–90. 10.1016/j.stem.2016.02.016 26952870 PMC5299540

[3] Cugola FR, Fernandes IR, Russo FB, Freitas BC, Dias JL, Guimarães KP, et al. The Brazilian Zika virus strain causes birth defects in experimental models. Nature. 2016;534:267–71. 10.1038/nature18296 27279226 PMC4902174

[4] Driggers RW, Ho CY, Korhonen EM, Kuivanen S, Jääskeläinen AJ, Smura T, et al. Zika virus infection with prolonged maternal viremia and fetal brain abnormalities. N Engl J Med. 2016;374:2142–51. 10.1056/NEJMoa1601824 27028667

[5] Jurado KA, Simoni MK, Tang Z, Uraki R, Hwang J, Householder S, et al. Zika virus productively infects primary human placenta-specific macrophages. JCI Insight. 2016;1:e88461. 10.1172/jci.insight.88461 27595140 PMC5007065

[6] Runge-Ranzinger S, Morrison AC, Manrique-Saide P, Horstick O. Zika transmission patterns: a meta-review. Trop Med Int Health. 2019;24:523–9. 10.1111/tmi.13216 30771269

[7] Besnard M, Lastère S, Teissier A, Cao-Lormeau V, Musso D. Evidence of perinatal transmission of Zika virus, French Polynesia, December 2013 and February 2014. Euro Surveill. 2014;19:8–11. 10.2807/1560-7917.ES2014.19.13.20751 24721538

[8] Besnard M, Dub T, Gérardin P. Outcomes for 2 children after peripartum acquisition of zika virus infection, French Polynesia, 2013–2014. Emerg Infect Dis. 2017;23:1421–3. 10.3201/eid2308.170198 28514228 PMC5547815

[9] Tolosa N. Surveillance protocols in public health: Zika virus diseases [in Spanish]. 2nd ed. Bogotá (Colombia): Instituto Nacional de Salud; 2016.

[10] Bayley N. Bayley scales of infant and toddler development. 3rd ed. San Antonio (TX): Harcourt Assessment Inc.; 2006.

[11] Zaidi MB, Cedillo-Barron L, González Y Almeida ME, Garcia-Cordero J, Campos FD, Namorado-Tonix K, et al. Serological tests reveal significant cross-reactive human antibody responses to Zika and Dengue viruses in the Mexican population. Acta Trop. 2020;201:105201. 10.1016/j.actatropica.2019.105201 31562846

[12] Estofolete CF, Terzian ACB, Colombo TE, de Freitas Guimarães G, Ferraz HC, da Silva RA, et al. Co-infection between Zika and different Dengue serotypes during DENV outbreak in Brazil. J Infect Public Health. 2019;12:178–81. 10.1016/j.jiph.2018.09.007 30301701

[13] Yin X, Zhong X, Pan S. Vertical transmission of dengue infection: the first putative case reported in China. Rev Inst Med Trop São Paulo. 2016;58:90. 10.1590/s1678-9946201658090 27982356 PMC5147720

[14] Brasil P, Pereira JP Jr, Moreira ME, Ribeiro Nogueira RM, Damasceno L, Wakimoto M, et al. Zika virus infection in pregnant women in Rio de Janeiro. N Engl J Med. 2016;375:2321–34. 10.1056/NEJMoa1602412 26943629 PMC5323261

[15] Ades AE, Soriano-Arandes A, Alarcon A, Bonfante F, Thorne C, Peckham CS, et al. Vertical transmission of Zika virus and its outcomes: a Bayesian synthesis of prospective studies. Lancet Infect Dis. 2021;21:537–45. 10.1016/S1473-3099(20)30432-1 33068528 PMC7992034

